# MTBHsp70-exFPR1-pulsed Dendritic Cells Enhance the Immune Response against Cervical Cancer

**DOI:** 10.7150/jca.29779

**Published:** 2019-10-19

**Authors:** Guangming Cao, Ran Cui, Chongdong Liu, Guyu Zhang, Zhenyu Zhang

**Affiliations:** Department of Obstetrics and Gynecology, Beijing Chao-Yang Hospital, Capital Medical University

**Keywords:** Cervical cancer, Immunotherapy, Dendritic cells, Recombinant antigen, FPR1

## Abstract

Cervical cancer is the most common malignancy of the female reproductive system. Dendritic cell (DC)-based immunological therapy is a novel treatment for this cancer. DCs are specialized antigen-presenting cells (APCs) in the human immune system, and they can activate the T cells used in tumor immunological therapy. In this study, we developed a novel immunotherapeutic peptide by linking the *Mycobacterium tuberculosis* (MTB) heat shock protein 70 (Hsp70) functional peptide to the extracellular domain of FPR1, a protein overexpressed in cervical cancer, to obtain an MTBHsp70-exFPR1 fusion protein. Our experiments confirmed that the MTBHsp70-exFPR1 protein could promote DC maturation and induce the secretion of IL-12p70, IL-1β, and TNF-α. The antitumor effect of human cytotoxic T lymphocytes (CTLs) activated by autologous DCs was assessed in NOG mice. These results indicate that DCs pulsed with MTBHsp70-exFPR1 can enhance antitumor immunity against cervical cancer, providing a novel immune therapeutic strategy.

## Introduction

Cervical cancer is the most common malignancy of the female reproductive system, and the incidence of cervical cancer is second only to that of breast cancer^1^. With the development and popularization of diagnosis and treatment technology, the incidence and mortality of cervical cancer have decreased by nearly 70%^2^, ^3^. However, in China, cervical cancer is still a high-risk disease among gynecological tumors^4^. At present, exploring the biological markers and therapeutic targets of cervical cancer remains the focus of diagnosis and treatment.

With the development of tumor immunology, immunotherapy now provides a fourth treatment mode after surgery, chemotherapy and radiotherapy^5^-^7^. Peptide vaccines typically utilize immunogenic epitopes from overexpressed tumor proteins (tumor-associated antigens or TAAs) for presentation to antigen-presenting cells (APCs)^8^. In recent years, an increasing number of Hsps have been used in tumor vaccines, such as Hsp27, Hsp65, Hsp70, Hsp90, Gp96, Hsp110, and Grp170. Hsp70 plays a dual role in cancer cells, as do many other molecular chaperones^9^. These studies have provoked the development of many anticancer vaccines based on the ability of Hsp70 to bind TAAs and to process them in APCs; samples from individual cancers were used to immunize patients^10^.

In this study, we chose to examine a *Mycobacterium tuberculosis* HSP70 (MTBHsp70) fusion with FPR1, which is overexpressed in cervical cancer^11^. Many immunological studies have demonstrated that MTBHsp70 has significant immunopotency that contributes to adaptive immune response. In addition, MTBHsp70 promotes antigen phagocytosis through binding to key receptors such as CD40 and CD91^12^. After uptake by APCs, HSPs can facilitate the presentation of complexed antigens by both class I and class II MHC receptors^13^. In vivo study has suggested that MTBHsp70 indeed provides protection against the effects of autoimmune diseases 14.

Formyl peptide receptor 1 (FPR1) is a G protein-coupled 7-transmembrane cell surface receptor (GPCR) involved in inflammation, wound healing and antimicrobial host defense^15^-^18^. However, the role of FPR1 in tumorigenesis remains poorly understood. In our previous study, we found that FPR1 is upregulated in cervical carcinoma tissues compared with peritumoral tissues by use of tissue microarray analysis (Guangming Cao's data are shown in another paper under review). These results suggested that FPR1 is involved in cervical carcinoma progression, but the molecular mechanism remains unclear. In this study, we investigate the potential role of FPR1 in cervical cancer immunotherapy.

In this study, we constructed recombinant proteins by fusing the extracellular domain of FPR1 (exFPR1) to the C terminus of MTBHsp70 with a GGGGS linker. Then, we investigated the immunotherapeutic effect of the MTBHsp70-exFPR1 fusion protein in cervical cancer therapy.

## Materials and methods

### Ethics statement

Our study using cord blood was approved by the ethics committee of Beijing Chaoyang Hospital, which is affiliated with the Capital Medical University. The collection and use of human cord blood samples, and informed consent was obtained from all the subjects. The methods were carried out in accordance with the approved guidelines.

### Expression, purification and analysis of exFPR1, MTBHsp70 and MTBHsp70-exFPR1 fusion proteins

We analyzed the protein sequences of MTBHsp70 (NP_214864.1) and human FPR1 (NP_001180235.1) firstly. The FPR1 protein contains 4 extracellular domains, which were the target domains in our study. The cDNA of MTBHsp70, the 4 extracellular domains of FPR1 (exFPR1) and the combined MTBHsp70-exFPR1 sequences were generated with a DNA synthesizer (MerMade 192E; BioAutomation, West Irving, TX, USA). Then, all the genes involved in this study were subcloned into the pUC57 plasmid. All constructs were validated by DNA sequencing. The HEK293 cell line was used as the host expression system for all recombinant protein production. Cell supernatant was harvested and filtered by a 0.22 µm membrane. Next, we used the His Bind Purification kit (Novagen, No. 70239) to purify the recombinant proteins. The identity and purity of the recombinant proteins were determined by SDS-PAGE. The molecular weights of MTBHsp70, exFPR1 and MTBHsp70-exFPR1 were 70 kD, 23 kD and 93 kD, respectively. Protein concentrations were measured by the Bradford assay.

### Mice and cell lines

Forty 4- to 5-week-old female NOG (NOD/Shi-scid/IL-2R γ null) mice were purchased from the Central Institute for Experimental Animals (CIEA) through the Beijing Vital River Laboratory Animal Company, where they were bred under strictly pathogen-free conditions. Animal experiments were performed according to the Guide for the Care and Use of Laboratory Animals of the National Research Council. The cervical cancer cell lines (SiHa and HeLa cells) were obtained from the Medical Research Center of Beijing Chaoyang Hospital. SiHa cells and HeLa cells were cultured in Roswell Park Memorial Institute (RPMI) on 1640 medium (Gibco, Gaithersburg, MD, USA) supplemented with 10% fetal bovine serum (HyClone, Logan, UT, USA).

### DC and cytotoxic T lymphocyte (CTL) induction

Samples of 40-50 mL citron anticoagulant cord blood were obtained from full-term healthy pregnancies during cesarean sections and used to obtain mononuclear cells via lymphocyte separation medium (TBD, Tianjin, China), according to the manufacturer's instructions. Mononuclear cells were suspended in improved minimum essential medium (IMEM; Gibco) and cultured for 6 hours at 37 °C and 5% CO_2_. After incubation, 95% of suspended cells were T cells, which were collected and cultured in RPMI 1640 plus 300 U/mL recombinant human interleukin (IL)2 and 50 ng/mL purified anti-human CD3 monoclonal antibody (eBioscience, San Diego, CA, USA). Adherent cells could be differentiated into DCs through culture in IMEM containing 1000 U/mL recombinant human colony-stimulating factor 2 and 500 U/mL recombinant human IL4 (PeproTech, Rocky Hill, NJ, USA). On day 5, the DCs were loaded with 10 mg/mL recombinant MTBHsp70-exFPR1 fusion protein, a mixture of MTBHsp70 and exFPR1, MTBHsp70, exFPR1, or phosphate-buffered saline (PBS). On day 8, the DCs were collected for flow cytometry or cocultured with autologous lymphocytes for 7 days as therapeutic vaccines for further experiments.

### Cytokine detection by ELISA

ImDCs were treated with different recombinant proteins (500 ng), and then, the cell supernatants of 5 groups were harvested 72 hours later. The supernatant levels of IFN-12p70, TGF-β and IFN-γ were measured with human IFN12p70, TGF-β and IFN-γ ELISA kits (Neobioscience Technology Company) according to the manufacturer's instructions.

### Flow cytometry analysis

DCs were washed and incubated with FITC-conjugated antibody against either CD80 or CD83 or with PE-conjugated antibody against CD11c and HLA-DR (BD) for 30 min at room temperature. After washing with PBS, the DCs were fixed with 2% paraformaldehyde and analyzed by flow cytometry on a FACS Aria II (BD 16 Biosciences, Franklin Lakes, NJ, USA). We isolated human lymphocytes from mouse peripheral blood by use of lymphocyte separation fluid. Then, the cells were washed and incubated with PE-Cy5-conjugated human CD3, FITC-conjugated human CD4, and PE-conjugated human CD8 antibodies (BD) and detected by a Beckman Coulter Gallios (Brea, CA, USA).

### RNA extraction, reverse transcription and real-time PCR

Total RNA was extracted from the cells or the placental tissues using TRIzol (Invitrogen) following the manufacturer's instructions. Reverse transcription was performed with 2 μg RNA using a PrimeScript RT Reagent Kit with gDNA Eraser (Takara Bio Inc.). qRT-PCR was performed using a Roche Light Cycler 480 II detection system (Roche, Basel, Switzerland). The detection of cDNA was carried out following the instructions of the SYBR® Premix Ex TaqTM kit (Takara, Dalian, China), and the reaction for each sample was carried out in duplicate at 95 °C for 30 s, followed by 40 cycles of 95 °C for 5 s and 60 °C for 31 s. The relative expression level of FPR1 was normalized to the value of glyceraldehyde-3-phosphate dehydrogenase (GAPDH) The PCR primers used for real-time PCR were as follows: FPR1, 5-GCTCCTCACATTGCCAGTTAT-3′ (forward) and 5′-CGTTGGTCCAGGGCGAAAA-3′ (reverse); GAPDH, 5′-AAGGTCATCCCTGAGCTGAAC-3′ (forward) and 5′-ACGCCTGCTTCACCACCTTCT-3′ (reverse). The relative mRNA levels were calculated by the 2^-ΔCT^ method.

### Western blot

Aliquots of 30 μg proteins from the cellular extracts were loaded onto 10% acrylamide gels for SDS-PAGE. The proteins were separated and transferred to nitrocellulose membranes (Millipore, St Quentin-en-Yvelines, France), incubated with specific antibodies overnight, and then incubated with specific horseradish peroxidase (HRP)-labeled secondary antibodies. Films were scanned using a calibrated Bio-Rad GS 800 densitometer, and signals were analyzed using ImageJ. Anti-FPR1 and anti-GAPDH antibodies were obtained from Abcam.

### Cytotoxicity assay

To induce tumor-specific CTL, T lymphocytes were incubated with DC at a ratio of 10:1 in 24-well culture plates in RPMI 1640 medium containing IL-2 for 5 days at 37 °C and 5% CO_2_. The CTLs were harvested and incubated with target cells (SiHa cells and HeLa cells) at ratios of 2.5:1, 5:1, 10:1, and 20:1 in 96-microwell plates at 37 °C and 5% CO_2_ for 72 h as the reactive cell group. Meanwhile, target cell, CTL and culture solution blank groups were established as control groups. In the last 4 h, 20 μL CCK8 solution was added. The absorbance OD value was detected by an enzyme-labeled instrument at 450 nm. The following formula was used to calculate the killing rate: killing rate = [1-(OD value of reactive cell well-OD value of effector cell well)/ (OD value of target cell well)]×100%.

### In vivo tumor therapeutic assay

To assess the role of the in vivo tumor therapeutic effect on cervical cancer development, five groups of NOG mice, with 6 animals in each group, were injected with SiHa cells (5×10^6^ per 100 μL) in the subcutaneous tissue of the upper back, and tumor diameters were measured with sliding calipers in two dimensions every 5 days. Each tumor-bearing mouse was immunized twice by tail vein injection with 5×10^6^ human lymphocytes stimulated by different proteins on Day 15 and Day 25. The time of test termination and organ/tissue sampling was determined by the tumor size in the control animals reaching a volume of 0.5 to 1 cm^3^. Tumor volume was calculated by the formula: Tumor volume = tumor length×tumor width^2^×0.5

### Immunohistochemistry and HE staining

After the experiments ended, organs and tumor tissues were isolated and fixed in paraformaldehyde for 24 h. Paraffin-embedded sections were stained with HE. The Ki67 and cleaved caspase 3 monoclonal antibody (Abcam) diluted 1:100 in 5% BSA was added. An HRP-labeled goat anti-rat IgG antibody was used as the secondary antibody, and staining was performed with a solution containing DAB.

### Statistical analysis

All statistical analyses were performed with GraphPad Prism 6.0 software (GraphPad Software Inc., La Jolla, CA, USA). Analysis of variance and Student's t-test were used to compare continuous variables. P values less than 0.05 were considered statistically significant.

## Results

### Expression and analysis of recombinant proteins

**Figure 1:**
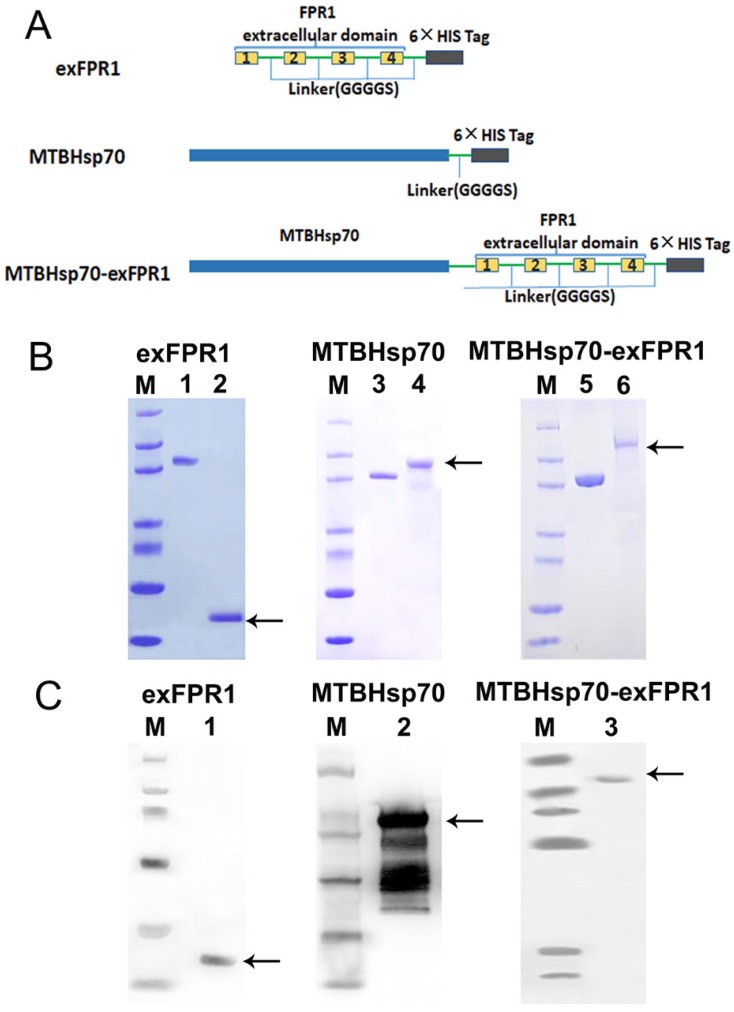
** Expression and analysis of recombinant proteins.** (A) Schematic representation of exFPR1, MTBHsp70 and the MTBHsp70-exFPR1 fusion protein. MtHsp70, *Mycobacterium tuberculosis* heat shock protein 70. ExFPR1, FPR1 extracellular domain. (B) SDS-PAGE analysis of exFPR1, MTBHsp70 and the MTBHsp70-exFPR1 fusion protein. M: protein marker; lanes 1, 3, 5: bovine serum albumin (1.5 mg); lane 2: exFPR1 protein; lane 4: MTBHsp70 protein; lane 6: MTBHsp70-exFPR1 fusion protein. Arrows show target proteins. (C) Western blotting analysis of exFPR1, MTBHsp70 and the MTBHsp70-exFPR1 fusion protein by anti-His-tag antibody. M: protein marker; lane 1: exFPR1 protein; lane 2: MTBHsp70 protein; lane 3: MTBHsp70-exFPR1 fusion protein. Arrows show target proteins.

We first analyzed the protein structure of FPR1. FPR1 contains 4 extracellular domains. As is well known, extracellular domains are the targets of immune recognition. To reduce ineffective epitopes, we constructed recombinant proteins by fusing the extracellular domain of FPR1 to the C terminus of MTBHsp70 with a GGGGS linker; the extracellular domain of FPR1 (exFPR1) and the MTBHsp70 protein were also included as controls in our study (Fig 1A). In addition, a 6×His tag flag protein was included at the C-terminal for use in purification (Fig 1A). The purity of the recombinant proteins was determined by SDS-PAGE gel with Coomassie blue staining (Fig 1B), and the results showed that the target band was single and could meet experimental requirements. The purified fusion protein was verified by Western blot analysis with an anti-6×His antibody and the molecular weights of the target bands were 70 kD, 23 kD and 93 kD (Fig 1C). The endotoxin concentration of recombinant protein involved in this study was <0.05 units/μg.

### MTBHsp70-exFPR1-induced DC maturation

**Figure 2:**
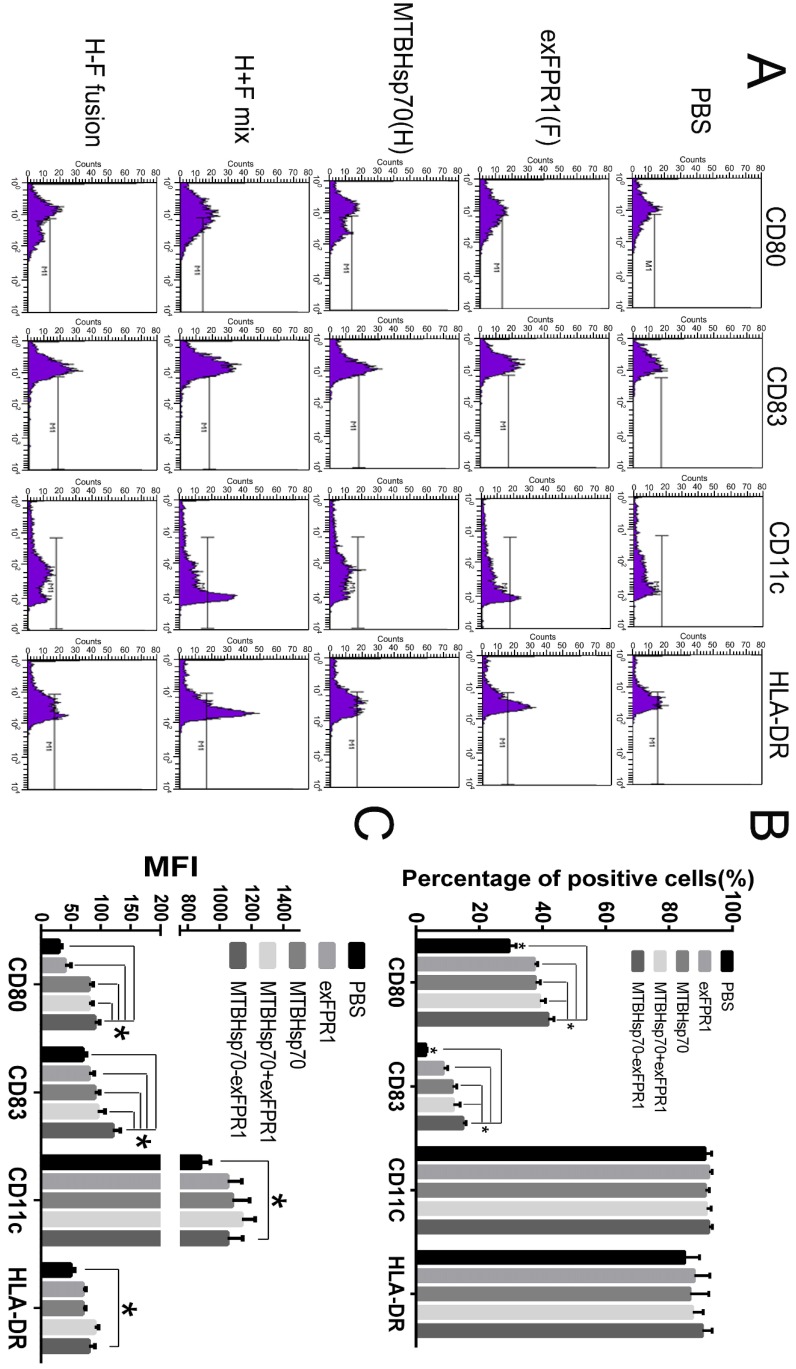
** MTBHsp70-exFPR1-induced DC maturation.** (A) Histograms showing the distribution of CD80, CD83, CD11c and HLA-DR cells in the different groups. Representative images from one of three individual experiments are shown. F indicates the exFPR1 group, H indicates the MTBHsp70 group, H+F mix indicates a simple mix of the exFPR1 and MTBHsp70 proteins, and H-F fusion indicates the MTBHsp70- exFPR1 fusion protein. (B) Percentages of positive cells for each DC surface marker in the different treatment groups. (C) Median fluorescence intensity (MFI) of each antibody signal in the different groups. Bar chart represents a statistical analysis based on the results of three independently repeated experiments. The data are expressed as the means ± SEM. *, p<0.05 compared with the corresponding data.

First, we investigated the effects of MTBHsp70, exFPR1 and the MTBHsp70-exFPR fusion protein during DC maturation. We induced monocytes isolated from umbilical cord blood to form dendritic cells. The mDCs were treated with or without 0.5 μg/mL of each protein at day 5. After 24 hours of incubation, the cells were stained with CD80, CD83, CD11c, and HLA-DR surface marker antibodies (Fig 2A). CD11c and HLA-DR were markers for DC purification. This purification resulted in more than 90% DC content in all groups, which met the requirements of the experiments. The effects of different proteins on the activation status of dendritic cells were observed. CD80 and CD83 were markers of mature DCs. Compared with that in the control group, all proteins used in our experiment could upregulate the expression of CD80 and CD83 in DCs (p<0.05), especially the fusion protein MTBHsp70-exFPR1 (Fig 2B), based on the median fluorescence intensity (MFI) of DC maturation markers CD80 and CD83. Notably, the exFPR1 protein alone could also promote DC maturation, but its effect was weaker than that of the MTBHsp70 protein. Overall, the MTBHsp70-exFPR1 fusion protein could significantly induce DC maturation.

### MTBHsp70-exFPR1 fusion protein promotes DC cytokine secretion

**Figure 3:**
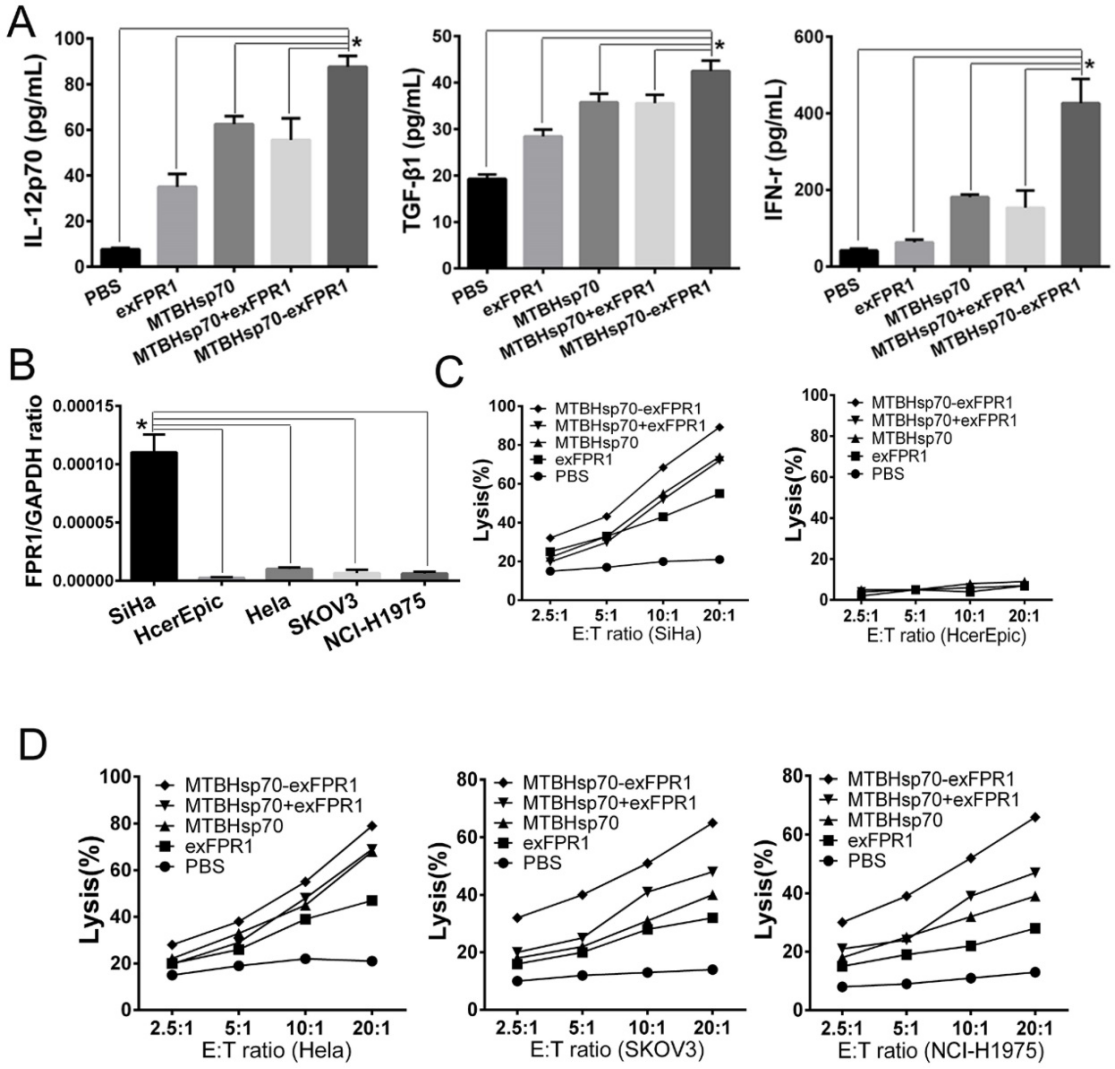
** MTBHsp70-exFPR1-induced DC cytokine production and DC-pulsed CTL cytotoxicity in vitro.** (A) IL-12p70, TGF-β1, and IFN-γ secretion in the supernatants of DCs stimulated with different antigens was tested by ELISA. (B) RT-PCR shows the relative expression of FPR1 in SiHa cells, HeLa cells, HcerEpic cells, SKOV3 cells and NCI-H1975 cells. (C-D) The lysis rate of the target cells was evaluated by an MTT assay. The bar chart represents a statistical analysis based on the results of three independently repeated experiments. The data are expressed as the means ± SEM. *, p<0.05 compared with the corresponding data.

We then determined by ELISA analysis that the MTBHsp70-exFPR1 fusion protein induces mDCs to secrete different cytokines. Consistent with the flow cytometry results, our results showed that all proteins used in our experiment could upregulate the concentrations of IFN-γ, TGF-β and IL-12p70 in DC culture supernatant, compared with that of the control group (Fig 3A). The exFPR1 protein alone could promote DC secretion of TGF-β and IL-12p70. MTBHsp70 and the MTBHsp70-exFPR1 fusion protein significantly promoted DC cytokine secretion (p<0.05). Thus, the MTBHsp70-exFPR1 fusion protein can promote functional DC maturation. These results indicated that the MTBHsp70-exFPR1 fusion protein may be involved in effector immune cell activation.

### MTBHsp70-exFPR1-pulsed DCs enhance the cytotoxic effects of CTLs

To test the efficacy of the CTLs treated with MTBHsp70-exFPR1-pulsed DC in target cell lysis, the lytic activity of the CTLs was measured. For this purpose, we cocultured protein-pulsed mDCs and T cells for 1 week and then performed a CTL assay. Cervical cancer cell lines (HeLa and SiHa cells), normal Human cervical epithelial cell line (HcerEpic cells), ovarian epithelial cell line (SKOV3 cells) and lung cancer cell line (NCI-H1975 cells) were used as targets to test whether the MTBHsp70-exFPR1-pulsed DCs could induce a specific T lymphocyte response in vitro. RT-PCR showed that all cells mentioned above expressed FPR1. In addition, SiHa cells expressed a higher level of FPR1 (Fig 3B). CTLs induced by protein-pulsed DC demonstrated specific cytotoxic activity toward cervical cancer cells compared with normal Human cervical epithelial cell line (HcerEpic cells) (Fig 3C). As shown in Fig 3D, compared with that of the control CTLs, significant differences in the killing effect toward cervical cancer cells were observed among different groups of CTLs at the same cell ratio. Compared with HeLa cells, SKOV3 cells and NCI-H1975 cells, CTLs activated by the MTBHsp70-exFPR1 fusion protein and exFPR1-pulsed DCs showed a high level of target cell killing toward SiHa cells. Thus, the cytotoxic effect was positively correlated with FPR1 expression level.

### Antitumor effect of CTLs activated by MTBHsp70-exFPR1-pulsed DCs in NOG mice

**Figure 4:**
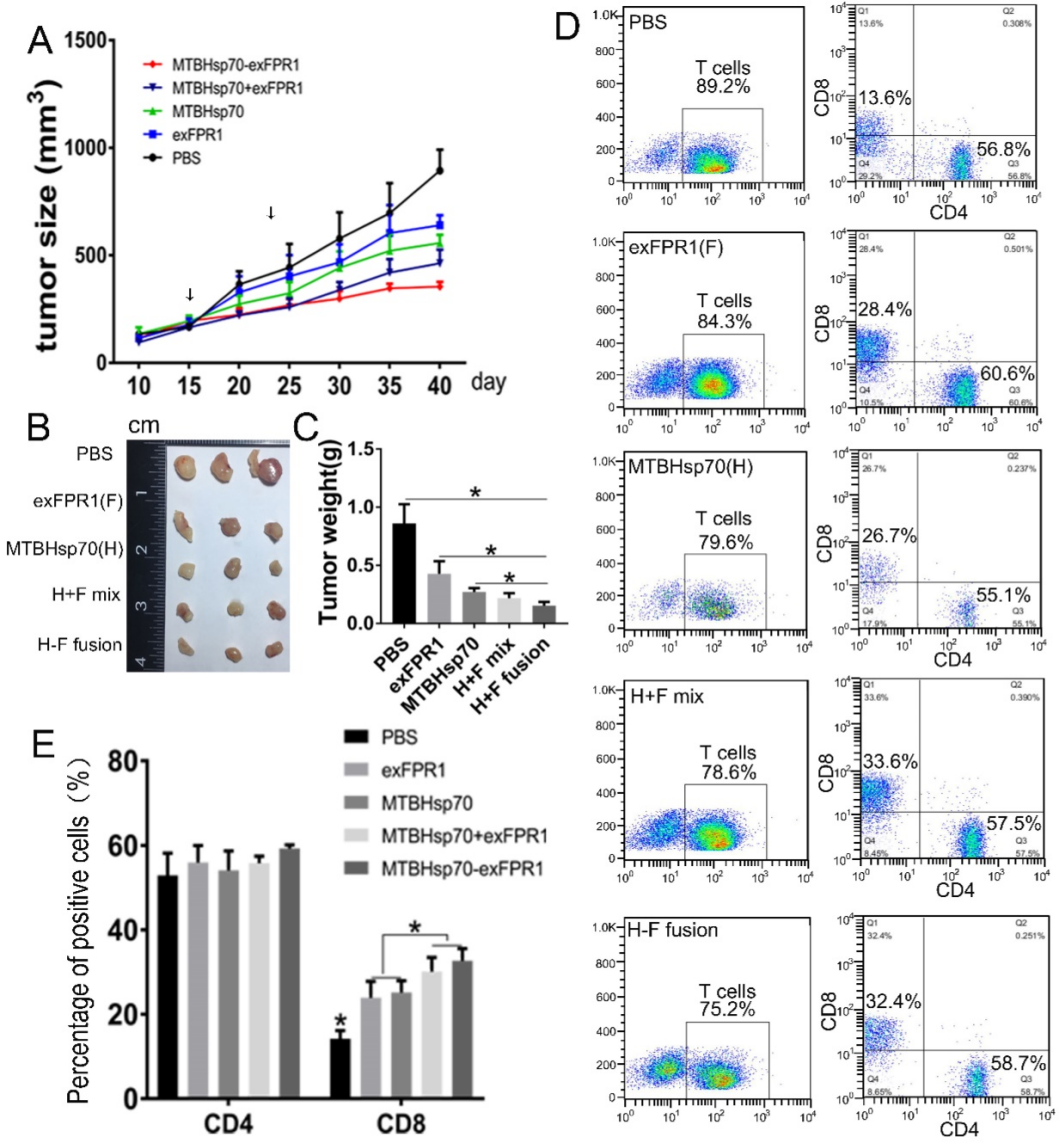
** Therapeutic effects of various protein-vaccine-loaded CTLs against cervical cancer cells in vivo.** (A) Tumor growth curve after different immune cells were injected through the tail vein. (B) Representative images of dissected tumors from each group on day 40. (C) Tumor weight in each treatment group. (D) Flow cytometry analysis of the percentage of human CD3+CD4+ and CD3+CD8+ T cells in the peripheral blood of treated mice. (E) Bar chart shows the percentages of human CD3+CD4+ and CD3+CD8+ T cells in the peripheral blood of treated mice. F indicates the exFPR1 group, H indicates the MTBHsp70 group, H+F mix indicates a simple mix of the exFPR1 and MTBHsp70 proteins, and H-F fusion indicates the MTBHsp70- exFPR1 fusion protein.

To test the ability of T cells activated by MTBHsp70-exFPR1-pulsed DCs to inhibit the growth of established tumors, NOG mice (six/group) were subcutaneously (s.c.) injected with 5×10^6^ cervical cancer cells. Fifteen days after tumor cell transplantation, each mouse formed a tumor with a diameter of 3-4 millimeters. Then, immune cells were injected through the tail vein. Ten days later, these mice were boosted with the same immune cells as in the first vaccination. Then, the mice were monitored every five days for tumor growth. Compared with those in the control group, the CTLs activated by all protein-pulsed DCs could inhibit tumor growth (Fig 4A). After immune cell injection, the inhibitory effect of MTBHsp70-exFPR1 was significantly stronger than that of MTBHsp70. We sampled the tumor when the experiments terminated, and the mean tumor volume and weight in the mice immunized with MTBHsp70-exFPR1 were significantly lower than those in the other treatment groups (Fig 4B, C). The peripheral blood lymphocytes of the mice were analyzed by flow cytometry. The results showed no difference in CD3+CD4+ T cell percentage among these groups. The percentages of CD3+CD8+ T cells in all the protein-pulsed DC groups were higher than that in the control group (p<0.05). The percentage of CD3+CD8+ T cells was similar between the MTBHsp70-exFPR1 and MTBHsp70+exFPR1 mixture groups, which were both higher than those in the MTBHsp70 and exFPR1 groups (Fig 4D, E).

### Biosecurity of CTLs activated by MTBHsp70-exFPR1-pulse d DCs in mice

**Figure 5:**
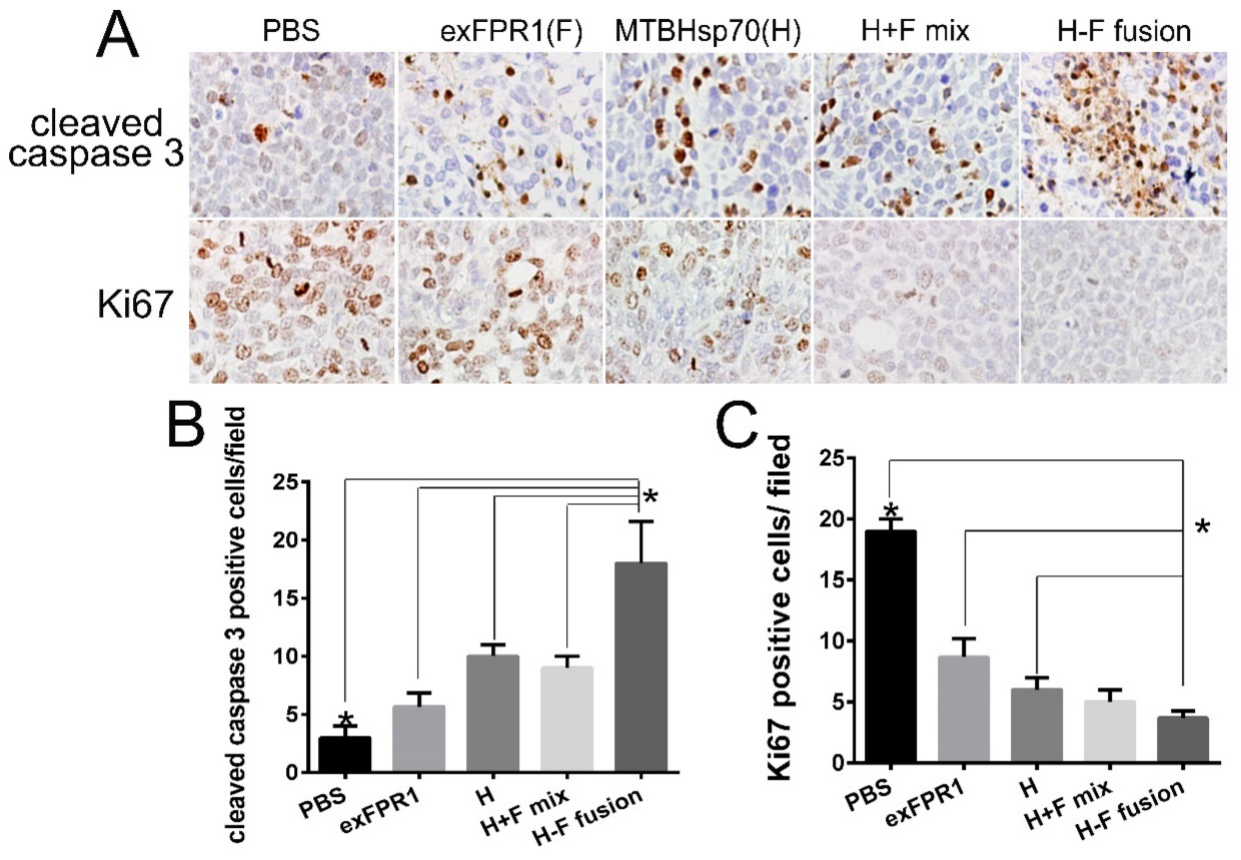
** HE and IHC analysis of tumor and major organs of NOG mice.** (A) HE staining analysis shows the structures major organs. (B) IHC analysis of the Ki67 and cleaved caspase 3 expression of tumor. (C) Bar chart shows number of cleaved caspase 3-positive cells per field. (D) Bar chart shows the number of Ki67-positive cells per field. Original magnification, 400×. The data are expressed as the means ± SEM. *, p<0.05 compared with corresponding data.

The biosecurity of immune therapy is a challenge for tumor therapy. To verify the safety of the CTLs activated by MTBHsp70-exFPR1-pulsed DCs in our experiments, we sampled major tissues and organs by hematoxylin-eosin (HE) staining and immunohistochemical (IHC) staining to determine their apoptosis and proliferation levels. In this experiment, we did not observe any abnormality of structure (Fig S1). However, significant changes in apoptosis and proliferation signals were detected in all tumor tissues treated with CTLs activated by protein-pulsed DCs (Fig 5A). CTLs activated by MTBHsp70-exFPR1-pulsed DCs significantly promoted tumor cell apoptosis compared to that in the other groups involved in our experiments (Fig 5B) (p<0.05). CTLs activated by MTBHsp70-exFPR1-pulsed DCs significantly inhibited tumor cell proliferation compared to that in the MTBHsp70 and exFPR1 groups (Fig 5C) (p<0.05).

## Discussion

Dendritic cells are the most powerful specialized APCs currently known^19^. They are the only APCs that can activate unsensitized initial T cells^20^. Immature dendritic cells (imDCs) have strong antigen capture and processing capacity, and mature dendritic cells (mDCs) stimulate the initial T cells involved in the immune response^20^, ^21^. In China, research on DC-based vaccines is still in the initial stage^22^-^24^. DC pulsing with crude extracts of tumor cells is a common treatment regimen, which induces antitumor effects of low strength and poor specificity and may even induce autoimmune diseases^25^-^27^. Safer options are urgently needed. The key point of preparation for DC vaccines is to explore the tumor-specific antigen peptides located at the cytomembrane.

Mycobacterial heat shock protein is very commonly used in the immunotherapy field^9^, ^10^, ^27^, ^28^. One of the most frequently used proteins in Hsp-based vaccines is Hsp70, which can be fused with TAAs to form new target proteins. FPR1 is a GPCR involved in inflammation, wound healing and antimicrobial host defense. As shown in our previous study, FPR1 is overexpressed in cervical cancer (Guangming Cao's data are shown in another paper under review). Thus, FPR1 is a potent therapeutic target that can be used in cervical cancer immunotherapy.

In our MTBHsp70-exFPR1-pulsed DC strategy, the MTBHsp70-exFPR1 fusion protein promotes DC phenotypic and functional maturation. Compared with that in the control group, all the proteins used in our experiment, especially the MTBHsp70-exFPR1 fusion protein, can upregulate the expression of CD80 and CD83 in DCs. Mycobacterial Hsp70 not only upregulates the expression of mature DC markers but also induces TGF-β1, IFN-γ, and IL-12 secretion by DCs. Therefore, HSP70 is an effective immune adjuvant that can be used in tumor immunotherapy. The exFPR1 protein alone induces a weaker DC maturation effect than the MTBHsp70 protein does, and the cytokine secretion levels in the different groups are consistent with their activation levels. Notably, cytotoxic effects are positively correlated with FPR1 expression levels. Thus, FPR1 is an effective immunotherapy target.

IL-12 is very important in promoting the polarization of Th1 cells and increasing the secretion of IFN-γ, IL-2 and TNF by CD8+ T cells, which is essential in the induction and maintenance of CTL. The IFN-γ secreted by Th1 cells or CD8+ CTLs has a powerful effect in enhancing the ability of DCs to produce IL-12, and IL-12 subsequently increases the production of IFN-γ. Our data demonstrate that the MTBHsp70-exFPR1 fusion protein induces DC maturation, perhaps through promoting cytokine secretion.

To further confirm the antitumor effect of the MTBHsp70-exFPR1 fusion protein in vivo, NOG mice, which are ideal hosts for the transplantation of human tumors, were chosen as a tumor model. MTBHsp70-exFPR1-pulsed DC-activated T cells inhibit tumor development by repressing tumor cell proliferation and promoting apoptosis, and this effect is caused by the cytotoxicity of CD8+ T cells. The cytokines secreted by DC and T cells repress tumor cell proliferation.

In summary, our study provides preclinical evidence that supports a protein-based immunotherapy that induces antitumor immune responses, which normally require DC-based approaches. Our research indicates that the MTBHsp70-exFPR1 fusion protein promotes DC maturation and mDC secretion of IFN-γ, TGF-β and IL-12p70. FPR1 is an effective immunotherapy target. MTBHsp70 exFPR1, as an antigen, can be used in cervical cancer immunotherapy.

## Supplementary Material

Supplementary figure S1.

## Abbreviations

DC: Dendritic cell
APCs: Antigen-presenting cells
MTB: Mycobacterium tuberculosis
Hsp70: Heat shock protein 70
FPR1: Formyl peptide receptor 1
CTL: cytotoxic T lymphocyte
NOG mice: NOD/Shi-scid/IL-2R γ null mice
