# Supplementary material for: MTBHsp70-exFPR1-pulsed Dendritic Cells Enhance the Immune Response against Cervical Cancer
Source: J Cancer. 2019 Oct 19;10(25):6364–73. doi: 10.7150/jca.29779 (PMC6856742; doi:10.7150/jca.29779)
Supplement: Supplementary file 1 — Supplementary figure S1. [file jcav10p6364s1.pdf]

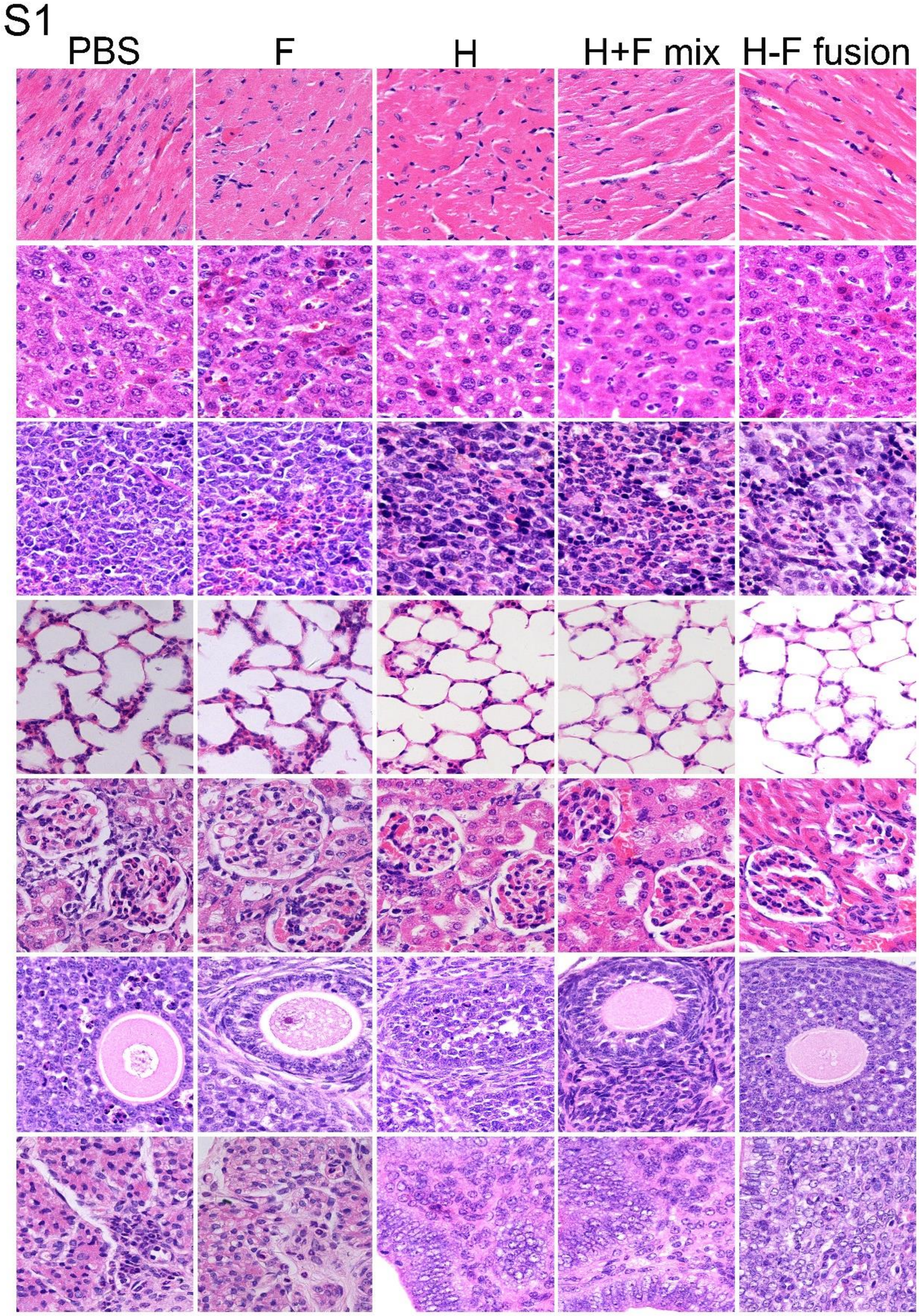

Fig S1. HE analysis of tumor and major organs of NOG mice. HE staining analysis shows the structures major organs.
